# Evidence-based programs for older persons in the Americas

**DOI:** 10.26633/RPSP.2021.91

**Published:** 2021-09-01

**Authors:** Patricia Morsch, Martha Pelaez, Enrique Vega, Carolina Hommes, Kate Lorig

**Affiliations:** 1 Pan American Health Organization Washington, D.C. United States of America Pan American Health Organization, Washington, D.C., United States of America; 2 Self-Management Resource Center, Aptos Calif. United States of America Self-Management Resource Center, Aptos, Calif., United States of America

**Keywords:** Self-management, self care, aged, delivery of health care, community health centers, Americas, Automanejo, autocuidado, anciano, prestación de atención de salud, centros comunitarios de salud, Américas, Autogestão, autocuidado, idoso, assistência à saúde, centros comunitários de saúde, América

## Abstract

In the current context of the aging of populations and the increase in multiple chronic conditions and dependence, it is important that health systems provide opportunities to improve capacities of older adults to enable healthy aging. Opportunities to enhance older adults’ abilities, including self-management, can be offered through evidence-based programs. Such programs have been proven effective in improving individuals’ symptoms and quality of life, often lowering health-care costs. Self-management evidence-based programs can foster the development of personal skills, increase confidence and motivation on self-care, and help individuals to make better decisions about their own health. This special report describes the implementation history of a self-management program in the Region of the Americas, and the barriers to and facilitators of implementation that can serve as examples for evidence-based program dissemination in the Region.

## RESUMEN

En el contexto actual de envejecimiento poblacional y aumento de la dependencia y de diversas enfermedades crónicas, es importante que los sistemas de salud brinden oportunidades para mejorar las capacidades de las personas mayores para propiciar un envejecimiento saludable. Las oportunidades de mejorar las capacidades de las personas mayores, incluido el autocuidado, se pueden ofrecer mediante programas basados en la evidencia. Estos programas han resultado eficaces para mejorar y la calidad de vida y los síntomas de las personas y, a menudo, para reducir los costos de salud. Los programas de autocuidado basados en la evidencia pueden fomentar el desarrollo de aptitudes personales, aumentar la confianza y la motivación sobre el autocuidado y ayudar a las personas a tomar mejores decisiones sobre su propia salud. En este informe especial se describe el proceso de ejecución de un programa de autocuidado en la Región de las Américas, y los factores facilitadores y los obstáculos para la ejecución que pueden servir de ejemplo para la difusión de los programas basados en la evidencia en la Región.

## RESUMO

No contexto atual de envelhecimento populacional e o consequente aumento de diversas doenças crônicas e da dependência, é importante que os sistemas de saúde criem oportunidades para a melhora da capacidade funcional da pessoa idosa visando ao envelhecimento saudável. Programas desenvolvidos com base em evidências científicas podem ser oferecidos porque comprovadamente ajudam a melhorar os sintomas e a qualidade de vida da pessoa idosa, reduzindo os custos em saúde. Os programas com enfoque no autocuidado estimulam o desenvolvimento de habilidades pessoais, aumentam a confiança e a motivação das pessoas idosas no próprio cuidado e contribuem para que elas tomem melhores decisões sobre a própria saúde. Este informe especial apresenta um programa de autocuidado implementado na Região das Américas, com a descrição do processo de implementação, das barreiras e dos facilitadores – uma experiência que pode servir de exemplo para difundir programas com base em evidências científicas na região.

For nearly two decades, the World Health Organization (WHO) has included the terms “self-management,” “self-care,” and “self-management support” in its documents. Self-management is associated with activities conducted by individuals themselves so they can live well with chronic conditions and, more importantly, increase their own ability and confidence to deal with these (1). As self-management strengthens an individual’s belief in his or her capacity to execute specific behaviors, it improves self-efficacy. This confidence in the ability to have control over one’s own motivation, behavior, and social environment culminates in more opportunities to maintain capacities and develop/maintain a better health status and quality of life (2, 3). While many publications distinguish between self-management and self-care, this report considers the two terms interchangeable.

Self-management support is defined by the activities conducted by health staff to enable individuals to self-manage. This includes education and support as well as more specific tasks such as problem-solving strategies and goal setting (1). This type of strategy can be delivered by evidence-based interventions. WHO has defined *evidence-based* as using data and results from research to address the health of populations in their own contexts, including identifying causes and related factors to health needs, as well as best practices of health promotion (4). It is important to point out that there is a difference between programs based on evidence and those that are evidence-based. The Evidence-Based Leadership Council (EBLC) states that evidence-based community health programs have specific characteristics to guarantee their effectiveness, such as having been tested with rigorous scientific methods, translated to the community, and further disseminated to the general population. Therefore, for a program’s successful replication at the community level, it is crucial to deliver training and provide essential materials, such as administrative manuals, to guarantee its fidelity (5).

It should be noted that both self-management and self-management support refer to skills and confidence as key elements of both self-management (what the individual does for him or herself) and for self-management support (what health professionals and others provide for patients).

Self-management strategies have been recognized as an important part of primary care to address chronic diseases and stimulate secondary prevention, due to the high involvement of individuals on their own care. Research has shown that apart from the benefits for the individual’s health and well-being, self-management strategies can reduce the economic impact of the increase of chronic conditions and provide more sustainable health systems (6). This special report aims to describe the implementation of a self-management program in the Region of the Americas and the barriers to and facilitators of implementation that can serve as case examples for program dissemination in the Region.

## SELF-MANAGEMENT EVIDENCE-BASED PROGRAMS IN THE CONTEXT OF AGING POPULATIONS

Life expectancy has increased, averaging 73 years worldwide, 77 years in the Region of the Americas, 75 in Latin America and the Caribbean (LAC), and 79 in North America (7, 8). Additionally, life expectancy in the Americas at 60 years of age has been estimated at 21 years; 81% of people born in the Region will live until age 60, while 42% will live beyond 80 (9).

Even though individuals are living longer, there is no evidence to support the idea that they are living longer with better health. In fact, life expectancy has increased faster than healthy life expectancy for both men and women, which reflects an increase in the number of years in ill-health at birth and at age 60 (10). As our population ages, there is an increase in chronic illness, a decrease in functioning, and for those over 80, which is the age group that is increasing more rapidly, an increase in poverty (11). These facts require a change in health systems and in the way programs and services are delivered. Care should be person-centered, integrated, consider a life-course perspective, and driven to improve individuals’ abilities (12). Healthy aging does not mean that people need to be disease-free. Healthy aging means ensuring coordinated care that manages disease with a focus on individuals’ personal goals, reflecting the ultimate objective which is living well and optimizing functional ability and intrinsic capacity. Intrinsic capacity, which is all the mental and physical capacities that an individual has, is a key component of healthy aging, as it is based on the concept of maintaining functional ability; that is, being able to be and to do the things one values for as long as possible (11).

Older people are the most vulnerable to chronic and infectious illness, as has been seen during the COVID-19 pandemic (13). Chronic conditions demand day-to-day self-management for the 99% of the time people are not in direct health care, as while living in the community, individuals are making judgements and taking actions concerning their health. In other words, they are self-managing. Self-management can be an important tool to promote healthy aging and avoid the losses of functional ability associated with chronic conditions, which can ultimately lead to dependence. In LAC, care dependence affects 12% of people over age 60 and almost 27% of people over age 80; it means that more than 8 million older people in LAC are unable to independently perform at least one basic activity of daily living. In the next 30 years, long-term care for older people will become an increasingly pressing concern for LAC countries, which calls for a better preparation for the delivery of long-term care services (14) but also strategies to better address diseases associated with care dependence from a preventive perspective.

In the face of a growing older population and the concomitant growth of demand for chronic illness care, emergency preparedness, including COVID-19 prevention and care, and management of functional ability, our health systems are facing financial challenges. They are called upon to increase their capabilities for diagnosis, treatment, rehabilitation, medication, monitoring, hospitalization, and end-of-life care to fully address the population’s needs.

## CALL TO ACTION TO IMPLEMENT EVIDENCE-BASED PROGRAMS TO ACHIEVE HEALTHY AGING

Actions to address older people with chronic conditions and losses of intrinsic capacity are not that different from those for mothers raising young children. The difference is that most countries have in place extensive maternal and child health care, including well-child visits, community vaccination programs, lactation programs, community education, mothers’ clubs, and community health visitors. What is missing for older people is a similar system to support chronic disease management, falls prevention, exercise, healthful eating, and caregiving. Just as in maternal and child health, the individual and the community have a key role in containing health care costs and improving the quality of life of older populations.

The WHO 2015 Global Report on Aging and Health made a call to support self-management, consisting in providing key information, skills, and necessary tools to manage health conditions, avert complications, increase intrinsic capacity, and maintain the quality of life of older persons (11).

More recently, the WHO proposal for a Decade of Healthy Aging (2021–2030) highlights evidence-based interventions as an opportunity to strengthen programs and services to improve health literacy and self-management and increase the opportunities for physical activity, good nutrition, and oral health (15). The Decade of Healthy Aging baseline report (2020) claims that evidence-based practices can provide insights on how best to facilitate choice and autonomy for older people, including managing self-care and using health services (10).

There is a wide range of different evidence-based programs that can be implemented in community settings to improve older adults’ intrinsic capacity, depending on the local needs and resources for implementation. Each domain of intrinsic capacity has an important impact on the others. Additionally, individuals’ self-efficacy and ability in making decisions have a direct impact on their adherence to such programs, and self-management can impact all the domains of intrinsic capacity. Such programs have been proven effective in improving individuals’ symptoms and quality of life, and often lower health care costs. They are beginning to be implemented by countries around the world. Examples can be seen in Table 1.

This report will address the results and implementation of the Living Healthy: Chronic Disease Self-Management Program (CDSMP) in the Region of the Americas.

## CHRONIC DISEASE SELF-MANAGEMENT PROGRAM (CDSMP)

The CDSMP, developed at Stanford University, is one of the most widely implemented evidence-based programs. It was developed to provide self-management skills and confidence to people with one or more chronic conditions, including physical and mental disorders. The rationale for its development was that most people over the age of 40 have comorbid conditions (16) and that the skills needed to self-manage these conditions are similar (17). The CDSMP is a highly interactive workshop offered in small groups (of 10–14 people) in the community by trained leaders, 2.5 hours once a week for six weeks, for individuals with chronic conditions. Recently, it has been customized so it can be offered virtually (to accommodate for physical distancing during COVID-19) or through a self-guided tool kit (18).

**TABLE 1.** Evidence-based programs to improve older adults’ intrinsic capacity

Programs	Intrinsic capacity domain	Format/setting
Living Healthy: a chronic disease self-management program	Self-efficacy and self-management support	Group intervention in community settings
Vivifrail; Walk with Ease; Capable; OTAGO	Locomotor capacity	One-on-one interventions in community or clinical settings
Active Living Every Day; Enhance Fitness; Fit and Strong; Matter of Balance; Tai Chi for Arthritis/for Fall Prevention	Locomotor capacity	Group interventions in community settings
Program for Encouraging Active Rewarding Lives (PEARLS)	Psychological capacity	One-on-one interventions in community or clinical settings
The Finnish Geriatric Intervention Study to Prevent Cognitive Impairment and Disability (FINGER)	Cognitive capacity	One-on-one and group interventions in community or clinical settings

***Source***: PAHO. Portfolio of evidence-based interventions [Working paper]. The table was prepared by the authors based on published data and best evidence to support the selection of programs to be included.

Each session of the CDSMP is built on three major skills associated with self-management: action planning, problem solving, and decision making. The specific topics that are discussed and shared in the group workshops are: symptom management, including cognitive and psychological symptoms; exercising; healthy eating; communicating with health providers; and medication management (18).

The broad CDSMP literature demonstrates that the program promoted reductions in health care utilization, with a saving of US$ 300–400 per participant. Most of these savings were in reduced time in hospital, fewer emergency department visits, and reductions in symptoms (19). The CDSMP has also demonstrated effectiveness when offered to diverse language-speaking populations: Chinese, Greek, Italian (20), Spanish (21), Bangladeshi (22); and in various countries: Canada (23), Japan (24), Australia (25), Spain (26), and Mexico (27). Finally, CDSMP has demonstrated efficacy and effectiveness when delivered via the Internet (28, 29) and as a tool kit delivered by mail (30). The CDSMP has reached more than a million people worldwide, is offered in more than 30 countries, and is available in more than 15 languages (18). In the Americas, the program is implemented by community-based organizations in Argentina, Brazil, Canada, Chile, Mexico, Peru, Puerto Rico, Trinidad and Tobago, the United States of America, and in the Eastern Caribbean Countries (ECC). Some of the countries’ experiences of program implementation are described below.

## IMPLEMENTATION HISTORY IN THE REGION

The Pan American Health Organization (PAHO) adopted the implementation of evidence-based programs in the Region of the Americas, including the CDSMP, because of the broad literature that supports positive results for individuals and health care systems, adaptations available in different languages, and feasibility of implementation in different realities.

During the period 2017–2019, PAHO launched a series of pilot programs in the following countries, which demonstrated interest and had local support for program development: Argentina, along with Programa de Atención Médica Integral (PAMI); Brazil, with support of the Pontifícia Universidade Católica do Rio Grande do Sul; Chile, through the Ministry of Health and the Red de Servicios; Mexico, with support of the Instituto Nacional de Geriatría and the Facultad de Enfermería de la Universidad Autónoma de Tamaulipas (Tampico); Peru, through the Ministry of Health; and in the ECC, through the ministries of health.

In total, 204 self-management community workshops were offered, reaching more than 2 000 persons in three years (2017–2019). In all countries, the programs were offered with the support of PAHO, but changes in government personnel, attrition in the pool of trainers and leaders, and lack of appropriate budgets allocated for self-management support—as well as close-downs due to the COVID-19 pandemic—have created a significant slowdown in the institutionalization of self-management as part of chronic care in the Region.

Despite a compelling rationale and the evidence of both clinical and cost effectiveness, evidence-based programs are far from reaching their potential. The focus on medical treatment rather than on public health approaches to secondary prevention, and the lack of focus on maintaining function and quality of life for older persons and persons with multiple chronic conditions, has made the adoption of self-management programs more difficult in the Region. In this section we highlight the experiences from a selected number of countries. Canada and the United States of America adopted the delivery of self-management support and education as a strategy for persons with chronic conditions much earlier than LAC (31, 32).

### Chile

During 2015–2016, PAHO approached the Ministry of Health of Chile with an introduction to the program and it was decided that the initial pilot would be in one of the health districts of Santiago. In 2017, the health district funded the training of leaders and master trainers as well as some of the program material. However, each municipality in the district was responsible for program coordination as well as participant recruitment and some of the didactic materials needed for program delivery.

Because the pressures in the health centers for delivering health care were great, the regional team began working with community-based partners such as community development officers and the mayor’s office in charge of aging, as well as the División de Organizaciones Sociales, to expand implementation outside of traditional health care settings.

#### Facilitators of implementation

- A health district director fully committed to the program.
- Dedicated, paid staff that increased as the program grew.
- The program was led by someone with a chronic condition who found the program personally useful and was committed to sharing this with others.
- Physicians and other health professionals trained as master trainers so that they could share the program with their professional colleagues and act as champions.
- The city mayor declared interest in the program and committed to it by designating a responsible person from the city council.

### ECC: Anguilla, Antigua and Barbuda, Barbados, British Virgin Islands, Dominica, Grenada, Montserrat, Saint Kitts and Nevis, Saint Lucia, Saint Vincent and the Grenadines

In 2017, PAHO invited two participants from each country to participate in a master trainer training. Each pair of trainers made a plan for implementation, including targeted populations, age, timeline, budget, etc. The program became part of the noncommunicable diseases program in the ministry of health. Primary health care providers identified people living with chronic conditions and referred them to the program. Additionally, efforts were made to ensure that both health professionals and nongovernmental organizations received information about the program.

#### Facilitators of implementation

- Strong support from the PAHO regional and PAHO ECC Office coordinators.
- Having one master trainer coordinator in each country. Country coordinators liaise closely with Caribbean (PAHO ECC Office) and regional coordinators.

### Mexico and Peru

The most successful implementation, both in Mexico and Peru, was led by two nursing faculty members from Tampico, Mexico, who worked closely with local health districts and with the ministries of health. With support from the university they created La Red de Automanejo (The Self-management Network) (27). The Network recruited and trained 32 self-management leaders working in community health centers. In 2014, the Network was able to obtain funding from the Government of Mexico’s Consejo Nacional de Ciencia y Tecnología (CONACYT), enhancing the Network with additional nursing schools in Mexico and Peru. Later the Network became part of the PAHO network.

#### Facilitators of implementation

- Funding from CONACYT provided both resources and support from health officials.
- The self-management program was a perfect fit with the mission of public health nurses working in the community doing health education. The new approach to supporting self-management was transformative and well accepted.
- Utilizing nurses already connected to the community health centers made the program sustainable, as it became an important tool for their work and did not represent an additional manpower expense.

### Lessons learned from the various pilots: barriers to implementation

Even though each country has its own system to follow, a lot of the barriers identified during program implementation were the same or very similar, and so they are presented together.

- The use of health care professionals as program leaders raised the cost of program delivery and resulted in disruption in continuity by frequent rotation of personnel from community health centers to hospital settings. In addition, it was difficult for some health professionals to understand the role of self-management and how it contributed to the control or improvement of chronic conditions.
- The use of volunteer lay leaders presented the challenge of maintaining a well-trained and motivated group of volunteers. A volunteer coordinator with time and skills for the nurturing of volunteers became essential for the success of the program.
- There was a lack of integration of self-management support programs in the menu of services provided by community health. The Chronic Care Model provides a clear justification for investment in activating patients as co-participants in health, but until this is reflected in the menu of services provided by the public health sector in collaboration with other community actors, the program funding and infrastructure will lack necessary resources.

## PUBLIC POLICY CONCLUSIONS AND RECOMMENDATIONS

With the changes in populations’ demographic and epidemiological profiles, policies that can provide long-term and sustainable results to favor healthy aging and management of chronic conditions are crucial. Providing tools for managing individuals’ health is not only an ethical imperative but also an economic one. Evidence-based programs, including those targeting self-management, can be an important resource to promote more years lived in good health, while reducing health care costs. From the implementation history of the self-management program in the Region, it is possible to highlight many facilitators of and barriers to implementation. However, more research is needed to identify the main program’s results in the Region, as well as experiences with other programs that can maintain or improve older adults’ intrinsic capacity and boost healthy aging.

## Disclaimer.

Authors hold sole responsibility for the views expressed in the manuscript, which may not necessarily reflect the opinion or policy of the *RPSP/PAJPH* and/or PAHO.

## References

[1] 1. Institute of Medicine; Board on Health Care Services. The 1st Annual Crossing the Quality Chasm Summit: A Focus on Communities: Report of a Summit. Washington, DC: The National Academies Press; 2004. 10.17226/11085 25009886

[2] 2. American Psychological Association [Internet]. Washington, DC: APA: 2009 [cited 2021 May 1]. Teaching Tip Sheet: Self-Efficacy. Available from: https://www.apa.org/pi/aids/resources/education/self-efficacy

[3] 3. Bandura A. Self-efficacy: the exercise of control. New York: Freeman; 1997.

[4] 4. Smith BJ, Tang KC, Nutbeam D. WHO Health Promotion Glossary: new terms. Health Promot Int. 2006 Jul 18;21(4):340–5. 10.1093/heapro/dal033 16963461

[5] 5. Evidence-Based Leadership Council [Internet]. Evidence-based programs. [No place]: EBLC; 2021 [cited 2021 May 1]. Available from: http://www.eblcprograms.org/evidence-based/what-is-eb/

[6] 6. Dineen-Griffin S, Garcia-Cardenas V, Williams K, Benrimoj SI. Helping patients help themselves: A systematic review of self-management support strategies in primary health care practice. PLoS One. 2019 Aug 1;14(8):e0220116. 10.1371/journal.pone.0220116 PMC6675068 31369582

[7] 7. World Health Organization [Internet]. Geneva: WHO; 2020 [cited 2021 May 1]. The Global Health Observatory. Available from: https://www.who.int/data/gho/data/indicators/indicator-details/GHO/life-expectancy-at-birth-(years)

[8] 8. The World Bank [Internet]. Washington, DC: World Bank; 2019 [cited 2021 Apr 5]. Data. Life expectancy at birth, total (years). Available from: https://data.worldbank.org/indicator/SP.DYN.LE00.IN

[9] 9. Pan American Health Organization. Health in the Americas +: Summary: Regional Outlook and Country Profiles. 2017 edition. Washington, DC: PAHO; 2017.

[10] 10. World Health Organization. Decade of Healthy Ageing: Baseline Report. Geneva: WHO; 2020. 220 p. Available from: https://www.who.int/publications/i/item/9789240017900

[11] 11. World Health Organization. World Report on Ageing and Health. Geneva: WHO; 2015.

[12] 12. World Health Organization. Integrated care for older people (ICOPE): Guidance on person-centred assessment and pathways in primary care. Geneva: WHO; 2019.

[13] 13. United Nations. Policy Brief: The Impact of COVID-19 on older persons. Copenhagen: United Nations; 2020 May. 16 p.

[14] 14. Bloeck MC, Galiani S, Ibarrarán P. Long-term care in Latin America and the Caribbean: Theory and policy considerations. Economía. 20(1):1–32. Available from: https://www.jstor.org/stable/26849048

[15] 15. World Health Organization. Decade of Healthy Ageing Final Proposal. Geneva: WHO; 2020. 1–24 p. Available from: https://www.who.int/initiatives/decade-of-healthy-ageing

[16] 16. Salive ME. Multimorbidity in Older Adults. Epidemiol Rev. 2013 Jan 1;35(1):75–83. 10.1093/epirev/mxs009 23372025

[17] 17. Clark NM, Becker MH, Janz NK, Lorig K, Rakowski W, Anderson L. Self-Management of Chronic Disease by Older Adults. J Aging Health. 1991 Feb;3(1):3–27. 10.1177/089826439100300101

[18] 18. Self-Management Resource Center [Internet]. Aptos, CA: SMRC; 2021 [cited 2021 May 1]. Chronic Disease Self-Management (CDSMP). Available from: www.selfmanagementresource.com

[19] 19. Lorig KR, Sobel DS, Stewart AL, Brown BW, Bandura A, Ritter P, et al. Evidence Suggesting That a Chronic Disease Self-Management Program Can Improve Health Status While Reducing Hospitalization. Med Care. 1999 Jan;37(1):5–14. Available from: http://journals.lww.com/00005650-199901000-00003 10.1097/00005650-199901000-00003 10413387

[20] 20. Swerissen H, Belfrage J, Weeks A, Jordan L, Walker C, Furler J, et al. A randomised control trial of a self-management program for people with a chronic illness from Vietnamese, Chinese, Italian and Greek backgrounds. Patient Educ Couns. 2006 Dec;64(1–3):360–8. 10.1016/j.pec.2006.04.003 16859871

[21] 21. Lorig KR, Ritter PL, González VM. Hispanic Chronic Disease Self-Management. Nurs Res. 2003 Nov;52(6):361–9. Available from: http://journals.lww.com/00006199-200311000-00003 10.1097/00006199-200311000-00003 14639082

[22] 22. Griffiths C, Motlib J, Azad A, Ramsay J, Eldridge S, Feder G, et al. Randomised controlled trial of a lay-led self-management programme for Bangladeshi patients with chronic disease. Br J Gen Pract. 2005 Nov;55(520):831–7. Available from: http://www.ncbi.nlm.nih.gov/pubmed/16281998 PMC1570787 16281998

[23] 23. McGowan P. The Efficacy of Diabetes Patient Education and Self-Management Education in Type 2 Diabetes. Can J Diabetes. 2011 Jan;35(1):46–53. 10.1016/S1499-2671(11)51008-1

[24] 24. Yukawa K, Yamazaki Y, Yonekura Y, Togari T, Abbott FK, Homma M, et al. Effectiveness of Chronic Disease Self-management Program in Japan: Preliminary report of a longitudinal study. Nurs Health Sci. 2010 Dec;12(4):456–63. 10.1111/j.1442-2018.2010.00559.x 21210924

[25] 25. Muscat DM, Song W, Cvejic E, Ting JHC, Medlin J, Nutbeam D. The Impact of the Chronic Disease Self-Management Program on Health Literacy: A Pre-Post Study Using a Multi-Dimensional Health Literacy Instrument. Int J Environ Res Public Health. 2019 Dec 19;17(1):58. 10.3390/ijerph17010058 PMC6982295 31861752

[26] 26. Gamboa Moreno E, Ochoa de Retana Garcia L, del Campo Pena ME, Sánchez Perez Á, Martinez Carazo C, Arbonies Ortiz JC, et al. A Pilot Study to Assess the Feasibility of the Spanish Diabetes Self-Management Program in the Basque Country. J Diabetes Res. 2016;2016:1–10. 10.1155/2016/9145673 PMC5227166 28119932

[27] 27. Córdova MIP de, Leon R, Gutierrez T, Mier N, Banda O, Delabra M. Effectiveness of a chronic disease self-management program in Mexico: A randomized controlled study. J Nurs Educ Pract. 2017 Feb 21;7(7):87. 10.5430/jnep.v7n7p87

[28] 28. Lorig KR, Ritter PL, Laurent DD, Plant K. Internet-Based Chronic Disease Self-Management. Med Care. 2006 Nov;44(11):964–71. 10.1097/01.mlr.0000233678.80203.c1 17063127

[29] 29. Lorig K, Ritter PL, Plant K, Laurent DD, Kelly P, Rowe S. The South Australia Health Chronic Disease Self-Management Internet Trial. Health Educ Behav. 2013 Feb 4;40(1):67–77. 10.1177/1090198112436969 22491008

[30] 30. Lorig K, Ritter PL, Moreland C, Laurent DD. Can a Box of Mailed Materials Achieve the Triple Aims of Health Care? The Mailed Chronic Disease Self-Management Tool Kit Study. Health Promot Pract. 2015 Sep 17;16(5):765–74. 10.1177/1524839915571633 25690615

[31] 31. Ory MG, Smith ML, Patton K, Lorig K, Zenker W, Whitelaw N. Self-Management at the Tipping Point: Reaching 100,000 Americans with Evidence-Based Programs. J Am Geriatr Soc. 2013 May;61(5):821–3. 10.1111/jgs.12239 23672545

[32] 32. Liddy C, Johnston S, Nash K, Irving H, Davidson R. Implementation and evolution of a regional chronic disease self-management program. Can J Public Heal. 2016 Mar 1;107(2):e194–201. 10.17269/cjph.107.5126 PMC6972270 27526218

